# Comprehensive Analysis of Secondary Metabolites in the Extracts from Different Lily Bulbs and Their Antioxidant Ability

**DOI:** 10.3390/antiox10101634

**Published:** 2021-10-17

**Authors:** Yu-Chao Tang, Yi-Jie Liu, Guo-Ren He, Yu-Wei Cao, Meng-Meng Bi, Meng Song, Pan-Pan Yang, Lei-Feng Xu, Jun Ming

**Affiliations:** 1 The Institute of Vegetables and Flowers, Chinese Academy of Agricultural Sciences, Beijing 100081, China; tangyuchao100@126.com (Y.-C.T.); liuyijie1209@163.com (Y.-J.L.); hgr0222@sina.com (G.-R.H.); caoyuwei1103@163.com (Y.-W.C.); bimengmenga@163.com (M.-M.B.); 15558111707@126.com (M.S.); yangpanpan@caas.cn (P.-P.Y.); xuleifeng@caas.cn (L.-F.X.); 2 College of Landscape Architecture and Forestry, Qingdao Agricultural University, Qingdao 266109, China

**Keywords:** genus *Lilium*, bulb extracts, phenolic acids, flavonoids, antioxidant activity, metabolites

## Abstract

The genus *Lilium* contains more than 100 wild species and numerous hybrid varieties. Some species of them have been used as medicine and food since ancient times. However, the research on the active components and the medical properties of lilies has only focused on a few species. In this study, the total phenolic acid content (TPC), total flavonoid content (TFC), and antioxidant capacity of 22 representative lilies were systematically investigated. The results showed that the TPC, TFC and antioxidant activity were highly variable among different lilies, but they were significantly positively correlated. Hierarchical cluster analysis indicated that *L. henryi* and *L. regale* were arranged in one group characterized by the highest TPC, TFC and antioxidant capacity, followed by Oriental hybrids and Trumpet and Oriental hybrids. The traditional edible and medicinal lilies were clustered in low TPC, TFC and antioxidant capacity group. A total of 577 secondary metabolites, including 201 flavonoids, 153 phenolic acids, were identified in the five species with great differences in antioxidant capacity by extensive targeted metabonomics. Differentially accumulated metabolites (DAMs) analysis reviewed that the DAMs were mainly enriched in secondary metabolic pathways such as isoflavonoid, folate, flavonoid, flavone, flavonol, phenylpropanoid, isoquinoline alkaloid biosynthesis, nicotinate and nicotinamide metabolism and so on. Correlation analysis identified that 64 metabolites were significantly positively correlated with antioxidant capacity (r ≥ 0.9 and *p* < 0.0001). These results suggested that the genus *Lilium* has great biodiversity in bioactive components. The data obtained greatly expand our knowledge of the bioactive constituents of *Lilium* spp. Additionally, it also highlights the potential application of *Lilium* plants as antioxidants, functional ingredients, cosmetic products and nutraceuticals.

## 1. Introduction

The genus *Lilium*, belonging to the family Liliaceae, is mainly distributed in temperate regions of the Northern Hemisphere including Eastern Asia, Europe, and North America [1]. There are approximately 115 species in this genus and 55 of them are native to China [2,3]. Due to their outstanding value as ornamental, edible, and medicinal plants, lilies are widely cultivated worldwide. Secondary metabolites of plants are important sources of many nutrients and natural medicines [4,5,6,7,8]. Plants of the genus *Lilium* are rich in chemical diversity and have been the focus of natural product chemistry research for decades. Phytochemical studies have shown that, in addition to essential primary metabolites, the chemical constituents of *Lilium* plants also include many secondary metabolites, including phenolic acids, flavonoids, saponins, sterols and alkaloids, which are important sources of medicinal compounds that can be used to treat diseases [9]. Among 115 wild species, over 30 have traditionally been documented to have historical ethnobotanical usages for disease treatment and health care [1,9,10]. *L. candidum* is used to promote wound healing in Europe and is used for treating burns and swelling in Japan [11,12]. *L. martagon* is used to treat liver disease in Europe [9,13]. According to traditional Chinese medicine (TCM), *Lilium lancifolium* Thunb., *Lilium brownii* F. E. Brown var. *viridulum* Baker and *Lilium pumilum* DC. are used medicinally for lasting cough, hemoptysis, anxiety, insomnia and dreaminess [14]. Moreover, the important and diverse biological activities of *Lilium* extract, such as anti-tumor [15], antioxidant [16], anti-inflammatory [17], antidepressant [18], hypoglycemic [16,19,20,21], have been reviewed by many modern pharmacological studies. In recent years, with the increasing interest in aesthetics, cosmetics have become popular. Lily extracts are also used as skin care substances in a variety of cosmetics [22,23].

Although more than 30 species of *Lilium* are used as traditional medicine or food worldwide, the research on the active components of *Lilium* is focused on only a few species, such as *L. lancifolium*, *L. davidii* var. *willmottiae*, *L. brownii* var. *viridulum*, *L. candidum* and *L. pumilum*, etc. [16,18,19,20,21]. Despite a long history of use, there are few systematic studies on the active components of different wild species of lily, and there are no reports on comparative studies among different varieties. In terms of the methods of detecting metabolites in *Lilium*, previous studies have either targeted qualitative and quantitative analysis of a few metabolites or non-targeted qualitative analysis of a large number of metabolites. These methods cannot take into account both the quantity of qualitative substances and the relative content of detected substances [16,18,19,20,21]. In fact, this genus has been given little scientific attention as functional and cosmetic products as well as nutraceuticals. Therefore, the identification of the chemical components of *Lilium* by new techniques on a large scale to maximize the utilization of *Lilium* plants is necessary [1,10]. Mass spectrometry coupled with high-performance liquid chromatography (HPLC–MS) has been increasingly used in the qualitative and quantitative analysis of complex matrices and has proven to be an effective tool to identify natural compounds due to the high sensitivity of mass spectrometry and the high separation ability of liquid chromatography [24]. Widely targeted metabonomics is different from the existing metabolite detection methods and integrates the advantages of nontargeted and targeted metabolite detection technologies. The obvious advantages of high throughput, high sensitivity, high qualitative accuracy, good repeatability, and complete database availability make it possible to qualitatively and quantitatively analyze many complex matrix materials with this method [25].

In this study, combined with physiological and biochemical experiments, we systematically studied the contents of phenolic acids and flavonoids, as well as their antioxidant activities of bulb extracts from different *Lilium* spp., including not only traditional edible and medical species, but also other wild species and commercial varieties of different hybrid sections. Furthermore, the secondary metabolites of five respective species with significant biological characteristic differences were profiled by extensive targeted metabonomic method and the differential accumulated metabolites among species were studied. Moreover, we analyzed the correlations between antioxidant capacity and metabolite accumulation. On the one hand, these results enrich our understanding of the chemical components of *Lilium* plants; on the other hand, they can provide a reference for the development of antioxidant and medicinal health resources from *Lilium* plants as well as increase the utilization of commercial lily varieties.

## 2. Materials and Methods

### 2.1. Material Collection and Preparation

In this study, 22 representative lily materials, including wild species and different hybrid sections, were collected from the Lily Germplasm Conservation Center of the Institute of Vegetables and Flowers, Chinese Academy of Agricultural Sciences, Xingyi, Guizhou, China (25°06′ N, 104°58′ E) in October 2020. Detailed information on the 22 chosen lilies is displayed in Table 1. To extract the active compounds, disease and insect-free scales of lily bulbs were selected and cleaned, and then freeze-dried by a vacuum freeze dryer (LGJ-25C, Beijing Sihuan Scientific Instrument Factory Co., Ltd., Beijing, China). The dried scales were ground and filtered through a 40-mesh sieve, and the prepared powder was stored at 4 °C in the dark until use.

### 2.2. Preparation of Methanol Extracts

The bioactive compounds were extracted by an ultrasound-assisted method using chromatographic grade methanol. In detail, 100 mg of each sample was weighed in a 10 mL sterile centrifuge tube, and 5 mL methanol was added. After shaking on a vortex oscillator, the extraction was carried out under 30 °C and 400 W ultrasonic conditions for 30 min by a numerically controlled ultrasonic cleaning instrument (UP4000HE, Nanjing Leijunda Ultrasonic Electronic Equipment Co., Ltd., Nanjing, China). The extracts were centrifuged (318K Labourzentrifugen, Sigma, Osterode, Germany) at 8000 rpm for 5 min at 25 °C. The extraction was repeated twice, and the combined supernatants were filtered through a 0.22 μm organic filter for determination of composition and antioxidant capacity.

### 2.3. Total Phenolic Acid and Total Flavonoid Content Determination

#### 2.3.1. Total Phenolic Acids

The total phenolic acid content of each extract was determined according to the method of Dowom, with some modifications, by using Folin–Ciocalteu reagent, and gallic acid was used as a standard [26]. Briefly, 400 μL methanol extract, 0.6 mL double distilled water and 0.25 mL 50% Folin–Ciocalteu were added to a 2 mL test tube, and the mixture was placed at room temperature for 2 min after mixing. Then, 750 μL 15% Na_2_CO_3_ were added and mixed well, and again, the mixture was placed at room temperature in the dark for 2 h. After incubation, 200 μL of reaction solution were added to a microplate for absorbance determination at 765 nm using a microplate reader (SpectraMax M2, Molecular Devices, Sunnyvale, CA, USA), and 400 μL of methanol were used as a reagent blank. Assays were performed after appropriate dilution for samples with high phenolic acid concentrations. The content of total phenolics in the samples was measured using a calibration equation (y = 0.0052 + 0.0318x, r^2^ = 0.9996) and expressed as mg of gallic acid equivalent per g of dry weight (GAE mg/g DW).

#### 2.3.2. Total Flavonoids

The total flavonoid content in each sample was measured according to a previously described spectrophotometric method with some modifications, and quercetin was used as a standard [27]. Briefly, 300 μL of the extract were mixed with 40 μL of a 5% NaNO_2_ solution in a test tube. After 6 min, 100 μL of a 10% AlCl_3_·6H_2_0 solution were added and allowed to stand for another 5 min before 400 μL of 1 M NaOH were added. The mixture was mixed well for each step. Two hundred microliters of the final mixture were added to the microplate for absorbance determination at 510 nm using a microplate reader (SpectraMax M2, Molecular Devices, Sunnyvale, CA, USA), and 300 μL methanol were used as a blank. The total flavonoid content in the samples was calculated by the standard curve equation (y = 0.0012x + 0.0023, r^2^ = 0.9990), and the results were expressed as mg quercetin equivalent per g of dry weight (QE mg/g DW).

### 2.4. Determination of Antioxidant Activity

#### 2.4.1. DPPH Radical Scavenging

The DPPH free radical scavenging ability was assessed according to the method of Jao, with some modifications [28,29], and trolox was used as standard. Briefly, 300 μL of the extract and 950 μL of 0.1 mM DPPH solution were added to a test tube and incubated for 30 min. Two hundred microliters of the final mixture were added to a microplate for absorbance determination at 517 nm using a microplate reader (SpectraMax M2, Molecular Devices, Sunnyvale, CA, USA). Assays were performed after appropriate dilution for samples with high antioxidant activity. The DPPH free radical scavenging ability of the samples was calculated by the standard curve equation (y = 0.0035x − 0.0151, r^2^ = 0.9962), and the results were expressed as μg trolox equivalent per g of dry weight (TE μg/g DW).

#### 2.4.2. Ferric Reducing Antioxidant Power (FRAP)

The ferric reducing ability was measured following previous methods with slight modifications [30], and trolox was used as the standard. Briefly, a 300 μL sample solution was mixed with 900 μL Fe^3+^-TPTZ working solution (10 mM solution of 2,4,6-tri(2-pyridyl)-S-triazine (TPTZ) in 40 mM HCl, 20 mM FeCl_3_·6H_2_O and volumes of acetate buffer pH 3.6). After 5 min of incubation, 200 μL of reaction solution were added to the microplate for absorbance determination at 593 nm using a microplate reader (SpectraMax M2, Molecular Devices, Sunnyvale, CA, USA), and 300 μL of methanol were used as a blank. Assays were performed after appropriate dilution for samples with high antioxidant ability. The FRAP in the samples was calculated by the standard curve equation (y = 0.0261x + 0.0248, r^2^ = 0.9991), and the results were expressed as μg trolox equivalent per g of dry weight (TE μg/g DW).

#### 2.4.3. Cupricion Reducing Capacity (CUPRAC)

The cupric ion reducing capacity was determined as described previously with slight changes [31]. Briefly, 100 μL extract or different concentrations of trolox, 0.5 mL of 10 mM CuSO_4_, and 0.5 mL of 7.5 mM neocuproine were added into a 2 mL test tube sequentially. After mixing well, the mixture was incubated for 30 min at room temperature. Then, 200 μL of the final mixture were added to the microplate for absorbance determination at 450 nm using a microplate reader (SpectraMax M2, Molecular Devices, Sunnyvale, CA, USA), and 100 μL methanol were used as a blank. Assays for samples with high antioxidant ability were performed after appropriate dilution. The CUPRAC of the samples was calculated by the standard curve equation (y = 0.003x + 0.1391, r^2^ = 0.9983), and the results were expressed as μg trolox equivalent per g of dry weight (TE μg/g DW).

### 2.5. Metabolite Profiling

#### 2.5.1. UPLC Conditions

The sample extracts were analyzed using an UPLC-ESI-MS/MS system (UPLC, SHIMADZU Nexera X2, www.shimadzu.com.cn/ (assessed on 21 April 2021); MS, Applied Biosystems 4500 Q TRAP, www.appliedbiosystems.com.cn/ (assessed on 21 April 2021)). The analytical conditions were as follows, UPLC: column, Agilent SB-C18 (1.8 µm, 2.1 mm × 100 mm); the mobile phase consisted of solvent A, pure water with 0.1% formic acid, and solvent B, acetonitrile with 0.1% formic acid. Sample measurements were performed with a gradient program that employed the starting conditions of 95% A, 5% B. Within 9 min, a linear gradient to 5% A, 95% B was programmed, and a composition of 5% A, 95% B was kept for 1 min. Subsequently, a composition of 95% A, 5.0% B was adjusted within 1.1 min and kept for 2.9 min. The flow velocity was set as 0.35 mL per minute; the column oven was set to 40 °C; the injection volume was 4 μL. The effluent was alternatively connected to an ESI-triple quadrupole-linear ion trap (QTRAP)-MS.

#### 2.5.2. ESI-Q TRAP-MS/MS

LIT and triple quadrupole (QQQ) scans were acquired on a triple quadrupole-linear ion trap mass spectrometer (Q TRAP), AB4500 Q TRAP UPLC/MS/MS System, equipped with an ESI Turbo Ion-Spray interface, operating in positive and negative ion mode and controlled by Analyst 1.6.3 software (AB Sciex). The ESI source operation parameters were as follows: ion source, turbo spray; source temperature 550 °C; ion spray voltage (IS) 5500 V (positive ion mode)/−4500 V (negative ion mode); ion source gas I (GSI), gas II (GSII), curtain gas (CUR) were set at 50, 60, and 25.0 psi, respectively; the collision-activated dissociation (CAD) was high. Instrument tuning and mass calibration were performed with 10 and 100 μmol/L polypropylene glycol solutions in QQQ and LIT modes, respectively. QQQ scans were acquired as MRM experiments with collision gas (nitrogen) set to medium. DP and CE for individual MRM transitions were performed with further DP and CE optimization. A specific set of MRM transitions was monitored for each period according to the metabolites eluted within this period.

### 2.6. Statistical Analysis

The TPC, TFC and antioxidant ability data of different lilies were sorted in Excel 2020 for Windows and analyzed in triplicate, and the results were expressed as the mean ± standard deviation (SD) using SPSS 16.0 for Windows. One-way analysis of variance (ANOVA) and Duncan’s multiple range tests were used to determine the significance of the differences among samples, with a significance level of 0.05. A two-tailed Pearson’s correlation test was performed to determine the correlations among mean values of different indexes, and hierarchical cluster analysis was used to group *Lilium* species.

For secondary metabolome data, significantly regulated metabolites between groups were determined by VIP ≥ 1 and absolute Log2FC (fold change) ≥ 1. VIP values were extracted from the OPLS-DA results, which also contained score plots and permutation plots and were generated using the R package MetaboAnalystR. The data were log transformed (log2) and mean centered before OPLS-DA. To avoid overfitting, a permutation test (200 permutations) was performed. The functions of DAMs were annotated based on the KEGG compound database to determine the metabolic pathways.

## 3. Results and Discussion

### 3.1. Total Phenolic Acid and Flavonoid Content

Due to their excellent antioxidant, bacteriostatic and anticancer functions, phenolics and flavonoids are among the most prevalent active components [32,33]. As secondary metabolites, phenolic acids and flavonoids can be extensively found in plant-derived foods, composing significant parts of our daily diet. The levels of TPC and TFC of 22 lily bulbs are shown in Table 2. The TPC was presented by GAE mg/g DW, and the TFC was presented by QE mg/g DW. The TPC of different lilies varies from *L.* ‘Jinghe’ (0.6 ± 0.07 GAE mg/g DW) to *L. regale* (13.73 ± 0.35 GAE mg/g DW), among which wild species *L. regale* and *L. henryi* stood out from all the tested lilies, followed by the O and OT types (2.83 ± 0.01~4.21 ± 0.07 GAE mg/g DW). The traditional edible and medicinal lily *L. lancifolium*, had a higher TPC of 2.25 ± 0.07 GAE mg/g DW than the others, and followed by the edible *L. davidii* var. *willmottiae* and *Lilium longiflorum* hybrid *L.* ‘White Heaven’. The remaining lilies had the lowest, having similar TPCs. The results showed that the TPCs of different genotype lilies varied greatly, which coincides with results from potato in which cultivar was the main variable affecting the biosynthesis of the studied phenolics [34]. The TFCs of the different lilies tested showed a similar trend with the TPCs, but with some differences. The TFCs of *L. regale* and *L. henryi* were 3–10 times higher than those of the other tested materials. Although both *L. lancifolium* and *L. brownii* var. *viridulum* are medicinal lilies, our results showed that there were significant differences in the contents of phenolic acids and flavonoids between them (Table 2).

For traditional edible lilies, *L. davidii*, *L. leichtlinii* var. *maximowiczii*, *L. davidii* var. *willmottiae*, *L. lancifolium* and *L. brownii* var. *viridulum*, *L. lancifolium* had the highest levels of both the TPC and TFC, and *L. brownii* var. *viridulum* had the lowest levels of TPC and TFC. There was no significant difference in the TFCs of *L. davidii*, *L. leichtlinii* var. *maximowiczii*, and *L. davidii* var. *willmottiae* (Table 2). This means that the nutritional and health effects of different lilies may be different. In a previous study, *L. regale* had higher contents of total phenolics, flavonoids and flavanols than the other five species (*L. concolor*, *L. pumilum*, *L. leucanthum*, *L. davidii* var. *unicolor* and *L. lancifolium*), consequently exhibiting the highest antioxidant ability [31]. We confirmed this result by the study of a broader selection of material, while our study also demonstrated that *L. henryi* is richer in TPC and TFC than other lilies except *L. regale*.

### 3.2. Antioxidant Activity

Due to the convenient and fast features of in vitro antioxidant activity assays, a variety of in vitro research methods have been widely used to evaluate the antioxidant activity of plant extracts [35]. Here, DPPH radical-scavenging activity, ferric reducing antioxidant power (FRAP), and cupric ion reducing capacity (CUPRAC) were used to determine the antioxidant ability of the extracts of different lilies [36]. Different concentrations of trolox were used to establish the standard curve for each test. The radical scavenging capacity (DPPH) and reducing power (FRAP and CUPRAC) of various extracts were expressed by the trolox equivalent antioxidant capacity (TE μg/g DW) in Table 2.

As shown in Table 2, the radical scavenging capacity of different lily extracts was significantly different. The best DPPH scavenging property was exhibited by the *L. regale* extract (16,707.07 ± 847.51 TE μg/g DW), which was 38 times as much as that of the lowest value observed for *L. brownii* var. *viridulum* (439.53 ± 171.03 TE μg/g DW). The second was *L. henryi* (13,249.75 ± 390.47 TE μg/g DW), being 30 times higher than the lowest value. The O and OT hybrids had moderate DPPH scavenging abilities from 2738.94 ± 265.84 to 4971.99 ± 402.33 TE μg/g DW, following a similar pattern as the TPC and TFC. For edible lily, *L. lancifolium* has the highest antioxidant capacity, although this value was not significantly different than that of *L. davidii* var. *willmottiae*. For the reducing power of FRAP and CUPRAC, *L. regale* (11,622.55 ± 344.81 and 32,304.44 ± 1878.93 TE μg/g DW, respectively) and *L. henryi* (8736.93 ± 645.45 and 26,593.33 ± 1205.54 TE μg/g DW, respectively) continued to show much higher levels than the other materials. In *Rhamnus petiolaris*, the contents of flavonols and anthocyanins were highly correlated with DPPH, and phenolic compounds were the main contributors to the antioxidant abilities of the members of the genus *Rhamnus* [37]. Our study confirmed that there were similar trends among the DPPH, FRAP and CUPRAC values and the TPC and TFC of the different lily bulb extracts, which means that the phenolic acids and flavonoids in lily bulbs might play vital roles in their antioxidant capacity. There were significant differences in TPC and TFC between *L. davidii* var. *willmottiae* and *L. lancifolium*, but there were no significant differences in antioxidant capacity in terms of DPPH, FRAP and CUPRAC values, which might be caused by the differences in antioxidant substances between them [38].

### 3.3. Correlation and Hierarchical Cluster Analysis of TPC, TFC and Antioxidant Capacity

The correlations among the TPC, TFC and antioxidant ability of DPPH, FRAP and CUPRAC were comprehensively analyzed based on the means of different tested indexes of each material. The results showed that all the tested factors were significantly highly correlated at the 0.01 level, and all the coefficients were above 0.977 (Table 3). The correlation coefficients between TPC and antioxidant indexes were 0.997 for DPPH, 0.992 for FRAP and 0.997 for CUPRAC, and the coefficients of TFC were 0.983, 0.986 and 0.977, respectively. This result indicated that although both TPC and TFC exhibited significant positive correlations with antioxidant capacity, TPC was more highly correlated with this capacity. The same pattern was also found in *Actinidia* extracts [34]. Our results revealed that the TPC was significantly related to the TFC, with a correlation coefficient of 0.977, in lily bulb extracts. This is reasonable since the biosynthesis of many flavonoids occurs downstream of phenolic acids in plants [39,40].

Previous studies suggested that the substances extracted by 80% methanol with 1 M HCl from lilies had no significant correlation with DPPH [31], but our results indicated that the TPC extracted by methanol was highly correlated with DPPH, which coincided with other reported results [38]. This means that the differences in substances extracted by different extraction methods from the same sample may cause divergences in antioxidant capacity [41]. Again, the significant correlation between methods was confirmed with three methods (DPPH, FRAP and CUPRAC). Above all, our results suggested that all the tested bulb extracts exhibited strong antioxidant activities, both in radical scavenging capacity and reducing power, and the antioxidant ability was generally positively correlated with the TPC and TFC.

**Figure 1:**
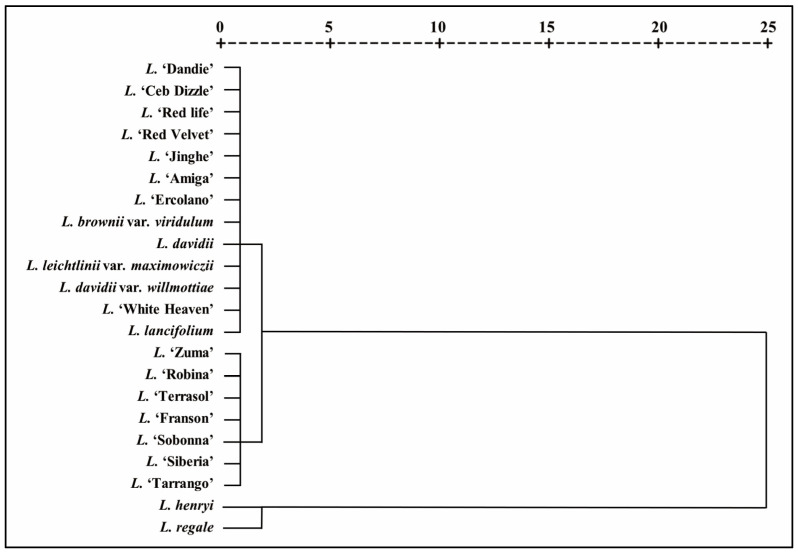
Dendrogram plot of hierarchical cluster analysis of 22 *Lilium* species based on the TPC, TFC and antioxidant properties.

Hierarchical cluster analysis is the most widely used method to classify the objects according to their characteristics [42]. Data on the TPC, TFC and antioxidant capacity (DPPH, FRAP and CUPRAC) were used to carry out hierarchical cluster analysis of the tested lilies, and 22 lilies were separated into three clusters, as shown in Figure 1. As the results showed, *L. regale* and *L. henryi* were clustered together with high TPC, TFC and antioxidant capacity, O and OT hybrid sections were arranged in one group characterized by moderate TPC, TFC and antioxidant properties, and the others, including traditional edible and medical lilies, belonged to a group with low TPC and TFC and weak antioxidant activity (Figure 1). The hierarchical clustering results showed the same change trend as the TPC, TFC and antioxidant capacity, which proved the reliability of our results above. These results suggested that, in addition to the traditional edible and medicinal lily, a large number of other species and cultivated varieties also have high antioxidant capacity, which can be used as important sources of natural antioxidants.

### 3.4. Profiling of Secondary Metabolites

The latest review summarized the information of 183 substances found in the genus *Lilium* thus far, including 95 saponins, 9 sterols, 23 phenylpropenoid glycerides, 10 alkaloids and 31 flavonoids, which is far from meeting the exploration on the medicinal function of lily. Therefore, it is necessary to identify the compounds contained in the genus *Lilium* more extensively [1]. Here, considering the results of TPC, TFC and antioxidant capacity, widely targeted metabolomics based on multiple reaction monitoring was used for qualitative and relative quantitative analysis of secondary metabolites in five representatively species (three traditional edible and medicinal lilies *L. davidii* var. *willmottiae* (LDW), *L. lancifolium* (LL), *L. brownii* var. *viridulum* (LBV), and two high TPC, TFC and antioxidant capacity species *L. henryi* (LH) and *L. regale* (LR)).

**Figure 2:**
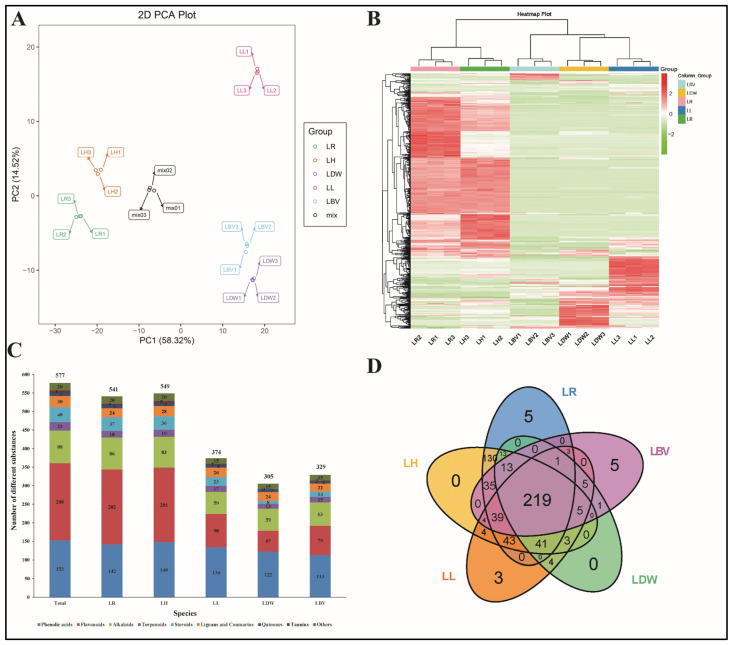
Overview of metabolites analysis detected in five lily species. LR: *L. regale*, LH: *L. henryi*, LL: *L. lancifolium*, LDW: *L. davidii* var. *willmottiae*, LBV: *L. brownii* var. *viridulum*. (**A**) Principal component analysis of the relative of metabolites for LR, LH, LL, LDW and LBV. (**B**) Cluster heatmap of metabolite content in different samples. (**C**) Distribution of substances in different materials. (**D**) Venn diagram of metabolite distribution in different materials.

**Figure 3:**
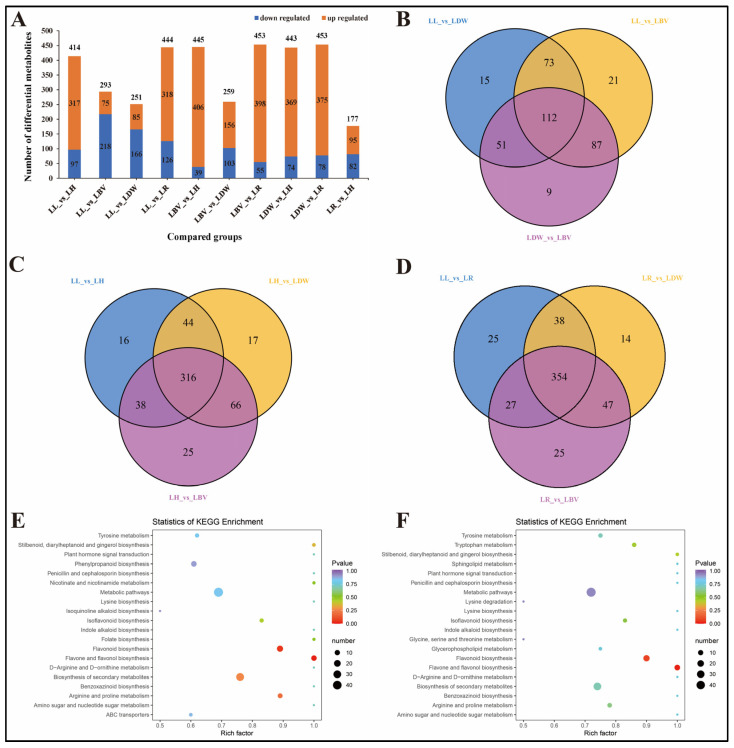
Differentially accumulated metabolites (DAMs) analysis. LR: *L. regale*, LH: *L. henryi*, LL: *L. lancifolium*, LDW: *L. davidii* var. *willmottiae*, LBV: *L. brownii* var. *viridulum*. (**A**) Bar graph of up- and downregulated DAMs from pairwise comparisons. (**B**) Venn graph for DAMs from the pairwise comparisons of traditional medicine and food used lilies (LL vs. LDW, LL vs. LBV and LDW vs. LBV). (**C**) Venn graph for DAMs from the pairwise comparisons between LH and three traditional medicine and food used lilies (LL vs. LH, LH vs. LBV and LH vs. LDW). (**D**) Venn graph for DAMs from the pairwise comparisons between LR and three traditional medicine and food used lilies (LL vs. LR, LR vs. LBV and LR vs. LDW). (**E**) Top 20 enriched KEGG pathways of DAMs in LL vs. LH. (**F**) Top 20 enriched KEGG pathways of DAMs in LL vs. LR.

Data validation and the results of total substance analysis are shown in Figure 2 or Figure S1. Unsupervised PCA (principal component analysis) was performed by statistics function prcomp within R (www.r-project.org) (Assessed on 21 April 2021). The correlation heat map between samples shows the high correlation within biological repeats and the differences between different species (Figure S2A). The differences among the five tested lilies were distinguished and verified by 2D PCA (Figure 2A) and 3D PCA (Figure S1B). The results showed that the first two principal components (PC1 and PC2) explained 72.84% of the overall variance (Figure 2A). Chinese traditional edible lilies LDW, LL and LBV were clearly separated from LH and LR in PC1 (58.32%), and LH and LR, LDW, and LBV and LL were clearly separated in PC2 (14.52%), respectively. LDW and LBV could be separated by PCA2 but the distinction was not obvious. The PCA highlighted a clear metabolic difference among the different *Lilium* species. In summary, the metabolic variation between LH, LR and LDW, LBV, LL was more obvious, whereas LH and LR, LDW and LL showed more similar metabolite profiling separately.

The cluster heatmap and class heatmap of the metabolites clearly showed the similarity of components among biological repeats and the difference of components amongst different species (Figure 2B or Figure S1C). A total of 577 secondary metabolites were target-identified, including 201 flavonoids, 153 phenolic acids, 88 alkaloids, 40 steroids, 30 lignans and coumarins, 23 terpenoids, 9 quinones, 6 tannins and 20 other compounds (Figure 2C). Detailed information on these substances is shown in Supplementary Table S1. Additionally, 541, 549 374, 305 and 329 compounds were detected in LR, LH, LL, LDW and LBV, respectively, and all the profiled metabolites were identified as nine main types (Figure 2C or Figure S1C). For instance, flavonoids and phenolic acids are the main secondary metabolites in lily, accounting for more than 50% of the total detected substances (Figure 2C or Figure S1D). Venn diagram analysis of the substances contained in the different species showed that 219 compounds were common to all five species, with few or no specific metabolites to a single species, indicating some similarity in metabolic composition among five tested species (Figure 2D). However, compared to the traditional edible lilies LDW, LBV and LL, LR and LH showed more numerous and higher contents of many substances (Figure 2B,C); LR and LH had 135 and 130 special compounds, respectively (Figure 2D).

The phenylpropane metabolic pathway involves the synthesis of many secondary metabolites, and most flavonoids are synthesized downstream of phenolic acids [39,40]. Since flavonoids and phenolic acids are the major antioxidants in plants [43] and account for the largest proportion of lily constituents (Figure 2C or Figure S2D), we performed correlation analysis for all flavonoids and phenolic acids detected in the five materials. The results indicated that most of the flavonoid have broad correlations with phenolic acid, and vice versa (Figure S1E). This result supports our findings of a significant positive correlation between the TPC and TFC (with a correlation coefficient of 0.977, Table 3) and provides new insights into the synergistic regulation of the biosynthesis and accumulation of phenolic acids and flavonoids in *Lilium* plants.

Difference analysis was performed for the substances detected from all samples, and compounds with fold change (FC) ≥ 2 or FC ≤ 0.5 and OPLS-DA VIP value ≥ 1 were defined as differentially accumulated metabolites (DAMs). The number of up/downregulated compounds resulting from pairwise comparison of tested species is shown in Figure 3A. The smallest number of differential metabolites was found in the LR vs. LH group with 177 (95 were upregulated and 82 were downregulated). Moreover, LH and LR had more differential metabolites than LDW, LBV, and LL, and among them, the substances whose contents were upregulated accounted for the majority (Figure 3A). A total of 112 differential metabolites were shared for three traditional edible species (LDW, LBV and LL), and LL was predominant compared with LDW and LBV not only in the total number of metabolites but also in the upregulated DAMs (Figure 3A,B). The metabolic comparative signatures among three traditional edible and medicinal lilies and the top 20 KEGG enrichment pathways of DAMs are presented in Supplementary Figure S2. Interestingly, LBV was dominant over LDW in the quantitative abundance of substances, but LDW contained more differentially upregulated metabolites than LBV (Figure 2C or Figure 3A). To explore the metabolite information in LH and LR, which had higher antioxidant activities and abundance and numbers of active compounds, we performed KEGG enrichment analysis of differential metabolites comparing them with those from Chinese traditional medicinal and edible lily LL. The top 20 most enriched pathways for the differential metabolites are shown in Figure 3E,F. Multiple amino acid metabolic pathways and secondary metabolic pathways (isoflavonoid biosynthesis, folate biosynthesis, flavonoid biosynthesis, flavone and flavonol biosynthesis, phenylpropanoid biosynthesis) were among them. These results may reveal the reason why the TPC and TFC were higher in LH and LR as well as their stronger antioxidant activities.

**Figure 4:**
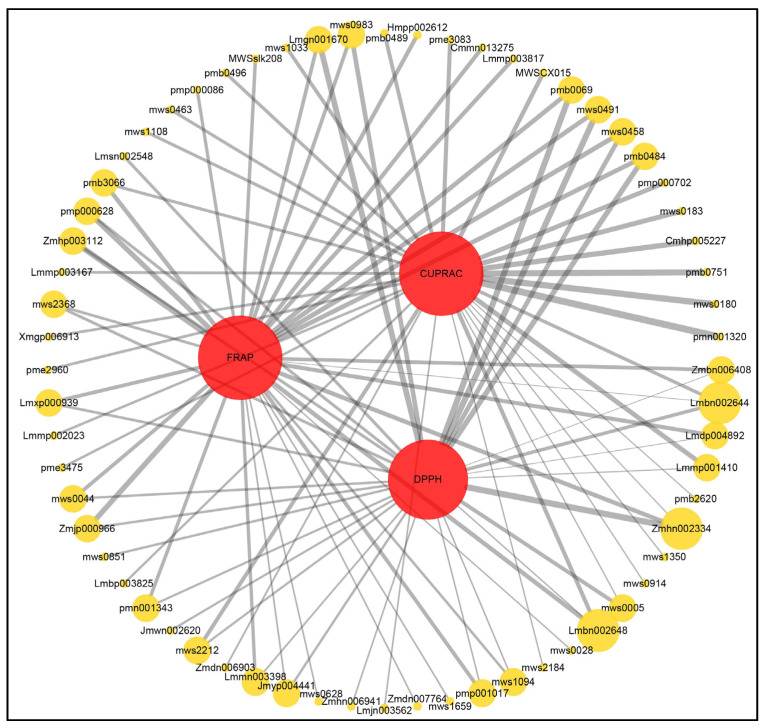
Network diagram between antioxidant capacity and metabolites. Red circles indicatedifferent antioxidant capacity (DPPH: 2,2-diphenyl-1-picrylhydrazyl radical scavenging ability; FRAP: ferric reducing antioxidant power; CUPRAC: cupricion reducing capacity.), yellow circles indicate different metabolites (The number above the circle indicates the component ID of Metware), and the line connecting the two circles represents the correlation, with the thicker the line the greater the correlation coefficient (r ≥ 0.9 and *p* < 0.0001).

To gain further insight into the types of antioxidants present in lily, we examined the correlation (spearman correlation coefficient) between the different metabolites detected and antioxidant capacity (DPPH, FRAP, CUPRAC), and identified 64 metabolites that were significantly positively correlated (r ≥ 0.9 and *p* < 0.0001) with antioxidant capacity. In which, 22 phenolic acid (vanillic acid-4-*O*-glucuronide, α-hydroxycinnamic acid, salicylic acid, 3,6′-diferuloylsucrose, 1-*O*-caffeoyl-β-d-glucose, vanillic acid, 2,5-dihydroxybenzoic acid, 3,4-dihydroxybenzoic acid, vanillin, 4-hydroxybenzaldehyde, syringaldehyde, ethyl caffeate, caffeic acid, tyrosol, benzamide, trans-5-*O*-(p-coumaroyl)shikimate, 3,4-dimethoxycinnamic acid, 5-*O*-p-coumaroylshikimic acid *O*-glucoside, 2-(formylamino)benzoic acid, 1-*O*-p-cumaroylglycerol, 1-feruloyl-sn-glycerol, 1-*O*-p-hydroxycinnamoyl-3-*O*-caffeoylglycerol, 6-*O*-feruloyl-d-glucose), 13 flavonoids (luteolin-7-*O*-gentiobioside, kaempferol-3-*O*-(6″-malonyl)galactoside, kaempferol-3-*O*-(6″-acetyl)glucoside, dihydroquercetin, hesperetin, pinobanksin, homoeriodictyol, dihydrokaempferol, naringenin chalcone, butin, 2,4,2″-tetrahydroxy-3′-prenylchalcone, safflor yellow A), 13 alkaloids (dihydrocaffeoylputrescine, caffeoylspermine, bis(caffeoyl)spermidine, zarzissine, tryptamine, phenethylamine, *N*-oleoylethanolamine, sophoridine, choline, *N*-glucosyl-p-coumaroylputrescine, *N*-feruloylagmatine, 9α-hydroxysophoramine, ebeinone) and 5 quinones (4,8-dihydroxy naphthol-1-*O*-glucoside, emodin, chrysophanol-8-*O*-glucoside, 6-methylaloe emodin, isoemodin), and 3 terpenoids (isopimaric acid, kaurenoic acid, 3’-*O*-d-glucosylgentiopicroside) were significantly positively correlated with at least one antioxidant capacity, suggesting that in addition to phenolic acids and flavonoids, some alkaloids and other substances in lily may also be important antioxidants (Figure 4, Table S2).

**Figure 5:**
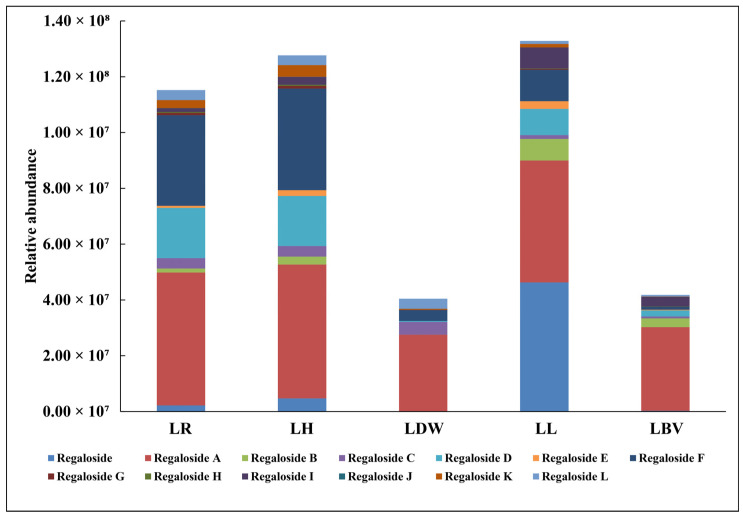
Distribution of phenylpropane glycerol glycosides (regalosides) in different lilies. LR: *L. regale*, LH: *L. henryi*, LL: *L. lancifolium*, LDW: *L. davidii* var. *willmottiae*, LBV: *L. brownii* var. *viridulum*.

Phenylpropanoid glycerol glucosides, also named as regalosides because they were first isolated from *Lilium regale*, are typical compounds in genus *Lilium* [44]. Studies have shown that regalosides have anti-oxidation, bacteriostasis, anti-inflammatory and other functions. Murray et al. [45] proved that phenylpropanoid glycerol glucosides can be used as an inhibitor of hepatic glucose production and may have potential to regulate metabolic syndrome and delay type II diabetes. The total glycosides of lily, including six regalosides, can protect cardiomyocytes by protecting mitochondria [46]. Regaloside I could be the main constituent in protecting human dermal fibroblasts from UVA-induced morphological changes [47]. In this study, 13 regalosides were detected in the five tested materials (Figure 5, Table S3). The results showed that the proportions of different regalosides varied in five lilies. In LR and LH, regaloside A, regaloside E and regaloside F accounted for a relatively high proportion; while, regaloside, regaloside A, regaloside B, regaloside D, regaloside F and regaloside I accounted for a relatively high proportion in LL; regaloside A had a higher proportion in all five tested lilies. These findings are of great value for the production of specific regalosides.

LDW, LL and LBV are Chinese traditional edible lilies, meanwhile, LL and LBV are used as TCM. At present, there is no specific description of the composition of the substances available for edible and medicinal lilies. The information on the composition of substances in lily that we obtained in the current study can provide a useful reference for the development of standards related to edible and medicinal lilies. The present results suggested that, besides traditional edible lilies, bulbs of the other lilies were also rich in phenolic acids, flavonoids and other bioactive ingredients, and had extensive antioxidant activity. Moreover, these characteristics of LR, LH, and O and OT hybrids were superior to those of the traditional edible lily, indicating the potential applications of a large number of other lily species and hybrids. Although some lilies have high TPC and TFC and strong antioxidant capacity, they also contain some substances that are not found in traditional edible and medicinal lilies. Considering the possible potential toxicity of different ingredients, even though there is almost no obvious acute toxic reaction to the human body, further studies on the constituents contained in different lilies are needed [1].

## 4. Conclusions

The TPC, TFC and antioxidant capacity (DPPH, FRAP and CUPRAC) in 22 representative lilies, including wild species and different hybrid sections, were systematically studied for the first time. *L. regale* and *L. henryi* have the highest TPC and TFC, leading to the highest antioxidant activity, followed by the O and OT hybrids, and the L, LA, A hybrids and the remaining wild species have the lowest TPC, TFC and antioxidant capacity. A total of 577 secondary metabolites, including 201 flavonoids and 153 phenolic acids, were profiled by extensively targeted metabolomics in five wild species (*L. davidii* var. *willmottiae*, *L. lancifolium*, *L. brownii* var. *viridulum*, *L. henryi* and *L. regale*). *L. regale* and *L. henryi* have great advantages in terms of both the quantity and content of active substances, leading to stronger antioxidant ability. A total of 64 compounds (including 22 phenolic acids, 13 flavonoids and 13 alkaloids) were identified to be significantly related to antioxidant capacity. Different species were all rich in phenylpropanoid glycerol glucosides, but the composition and content are quite different. These data greatly enriched our understanding of the active components of the genus *Lilium*. The present study reviewed the meaningful bioactive potential of traditional edible and medicinal lilies, as well as other wild and hybrid lilies, indicating that many wild and commercialized lilies can be also applied to obtain high added-value products, such as antioxidants, functional ingredients, cosmetic products and nutraceuticals.

## Figures and Tables

**Table 1:** Materials used in this study.

Number	Species/Cultivar	Genotype
1	*L. davidii*	Wild species (W)
2	*L. leichtlinii* var. *maximowiczii*	Wild species (W)
3	*L. davidii* var. *willmottiae*	Wild species (W)
4	*L. henryi*	Wild species (W)
5	*L. regale*	Wild species (W)
6	*L. lancifolium*	Wild species (W)
7	*L. brownii* var. *viridulum*	Wild species (W)
8	*L.* ‘Red Velvet’	Asiatic hybrids (A)
9	*L.* ‘Red life’	Asiatic hybrids (A)
10	*L.* ‘Dandie’	Asiatic hybrids (A)
11	*L.* ‘Jinghe’	Asiatic hybrids (A)
12	*L.* ‘White Heaven’	*L. longiflorum* hybrids (L)
13	*L.* ‘Amiga’	*L. longiflorum* and Asiatic hybrids (LA)
14	*L.* ‘Ceb Dizzle’	*L. longiflorum* and Asiatic hybrids (LA)
15	*L.* ‘Ercolano’	*L. longiflorum* and Asiatic hybrids (LA)
16	*L.* ‘Siberia’	Oriental hybrids (O)
17	*L.* ‘Sobonna’	Oriental hybrids (O)
18	*L.* ‘Tarrango’	Oriental hybrids (O)
19	*L.* ‘Zuma’	Oriental hybrids (O)
20	*L.* ‘Terrasol’	Trumpet and Oriental hybrids (OT)
21	*L.* ‘Robina’	Trumpet and Oriental hybrids (OT)
22	*L.* ‘Franson’	Trumpet and Oriental hybrids (OT)

**Table 2:** TPC, TFC and antioxidant activities of different lily extracts.

No.	Materials	Genotype	TPC (GAE mg/g DW)	TFC (QE mg/g DW)	DPPH (TE μg/g DW)	FRAP (TE μg/g DW)	CUPRAC (TE μg/g DW)
1	*L. davidii*	W	0.87 ± 0.04 jk	2.49 ± 0.70 efg	1047.84 ± 70.19 j	730.01 ± 53.82 i	2107.78 ± 416.78 h
2	*L. leichtlinii* var. *maximowiczii*	W	0.94 ± 0.07 jk	2.51 ± 0.88 efg	939.11 ± 141.34 jk	694.25 ± 17.56 ij	2018.89 ± 476.48 h
3	*L. davidii* var. *willmottiae*	W	1.88 ± 0.10 i	2.58 ± 0.49 efg	2057.96 ± 88.19 i	1071.01 ± 91.82 h	3818.89 ± 501.48 gh
4	*L. henryi*	W	11.63 ± 0.30 b	12.22 ± 0.99 b	13,249.75 ± 390.47 b	8736.93 ± 645.45 b	26,593.33 ± 1205.54 b
5	*L. regale*	W	13.73 ± 0.35 a	15.91 ± 2.78 a	16,707.07 ± 847.51 a	11,622.55 ± 344.81 a	32,304.44 ± 1878.93 a
6	*L. lancifolium*	W	2.25 ± 0.07 h	4.36 ± 0.29 cd	2242.67 ± 258.44 h i	1228.10 ± 58.15 gh	3227.22 ± 171.66 g
7	*L. brownii* var. *viridulum*	W	0.70 ± 0.09 kl	0.88 ± 0.18 h	439.53 ± 171.03 k	368.58 ± 37.74 j	1330.00 ± 57.74 h
8	*L.* ‘Red Velvet’	A	0.95 ± 0.07 jk	1.71 ± 0.60 fgh	1110.87 ± 44.55 j	691.70 ± 87.40 ij	1696.67 ± 624.50 h
9	*L.* ‘Red Life’	A	0.81 ± 0.02 kl	1.65 ± 0.34 fgh	947.55 ± 69.74 jk	579.31 ± 23.3 1 ij	1507.78 ± 157.53 h
10	*L.* ‘Dandie’	A	0.72 ± 0.08 kl	2.17 ± 0.57 fgh	1067.91 ± 289.86 j	676.37 ± 26.08 ij	1430.00 ± 272.85 h
11	*L.* ‘Jinghe’	A	0.60 ± 0.07 l	1.80 ± 0.39 fgh	696.17 ± 330.2 1 jk	428.61 ± 15.95 ij	1307.78 ± 221.94 h
12	*L.* ‘White Heaven’	L	1.82 ± 0.12 i	3.13 ± 0.25 def	2233.21 ± 255.68 hi	1054.41 ± 36.55 h	3585.56 ± 50.92 g
13	*L.* ‘Amiga’	LA	0.57 ± 0.07 l	1.65 ± 0.18 fgh	775.67 ± 19.11 jk	470.75 ± 11.71 ij	1374.44 ± 183.59 h
14	*L.* ‘Ceb Dizzle’	LA	1.11 ± 0.05 j	1.85 ± 0.55 fgh	1056.82 ± 291.92 j	584.42 ± 32.13 ij	1463.33 ± 317.98 h
15	*L.* ‘Ercolano’	LA	0.59 ± 0.06 l	1.31 ± 0.48 gh	692.71 ± 153.18 jk	415.84 ± 29.76 ij	1085.56 ± 320.3 h
16	*L.* ‘Siberia’	O	3.95 ± 0.41 d	4.73 ± 0.26 c	4721.04 ± 435.55 cd	3132.35 ± 89.85 c	8063.33 ± 416.33 d
17	*L.* ‘Sobonna’	O	3.21 ± 0.11 f	3.79 ± 0.38 cde	3459.84 ± 73.17 f	2106.21 ± 103.91 e	8118.89 ± 963.98 d
18	*L.* ‘Tarrango’	O	4.21 ± 0.07 c	5.19 ± 0.46 c	4971.99 ± 402.33 c	3119.28 ± 57.45 c	10,596.67 ± 352.77 c
19	*L.* ‘Zuma’	O	2.83 ± 0.01 g	2.83 ± 0.40 ef	3073.67 ± 184.01 fg	1707.52 ± 84.53 f	6041.11 ± 221.94 f
20	*L.* ‘Terrasol’	OT	3.65 ± 0.04 e	3.85 ± 0.06 cde	4238.72 ± 171.28 de	2426.47 ± 80.25 d	7996.67 ± 88.19 d
21	*L.* ‘Robina’	OT	3.16 ± 0.08 f	2.91 ± 0.27 ef	2738.94 ± 265.84 gh	1436.27 ± 93.52 fg	6674.44 ± 478.81 ef
22	*L.* ‘Franson’	OT	3.60 ± 0.16 e	2.48 ± 0.88 efg	4066.62 ± 234.24 e	2318.63 ± 191.36 de	7349.44 ± 359.62 de

TPC: total phenolic acid content; TFC: total flavonoid content; DPPH: 2,2-diphenyl-1-picrylhydrazyl radical scavenging ability; FRAP: ferric reducing antioxidant power; CUPRAC: cupricion reducing capacity; GAE: gallic acid equivalent; QE: quercetin equivalent; TE: trolox equivalent; W: wild species; A: Asiatic hybrids; L: *L. longiflorum* hybrids; LA: *L. longiflorum* and Asiatic hybrids; O: Oriental hybrids; OT: trumpet and Oriental hybrids; DW: dry weight. Values are expressed as the mean ± SD, on a dry basis. Different letters followed the numbers indicate statistically significant differences (*p* < 0.05).

**Table 3:** Correlation coefficients between TPC, TFC and antioxidant capacity.

	TPC	TFC	DPPH	FRAP	CUPRAC
TPC	1	0.977 **	0.997 **	0.992 **	0.997 **
TFC		1	0.983 **	0.986 **	0.977 **
DPPH			1	0.997 **	0.996 **
FRAP				1	0.993 **
CUPRAC					1

TPC: total phenolic acid content; TFC: total flavonoid content; DPPH: 2,2-diphenyl-1-picrylhydrazyl radical scavenging ability; FRAP: ferric reducing antioxidant power; CUPRAC: cupricion reducing capacity. ** Correlation is significant at the 0.01 level (2-tailed).

## Data Availability

Data within the article.

## Supplementary Materials

The following are available online at https://www.mdpi.com/article/10.3390/antiox10101634/s1, Figure S1: Distribution of metabolites in different samples. LR: *L. regale*, LH: *L. henryi*, LL: *L. lancifolium*, LDW: *L. davidii* var. *willmottiae*, LBV: *L. brownii* var. *viridulum*; Figure S2: Distribution of metabolites in different samples. LR: *L. regale*, LH: *L. henryi*, LL: *L. lancifolium*, LDW: *L. davidii* var. *willmottiae*, LBV: *L. brownii* var. *viridulum*; Table S1: Information on secondary metabolites in all samples; Table S2: Correlation coefficients and *p* values between metabolites and antioxidant capacity; Table S3: Phenylpropanoid glycerol glucosides (regalosides) detected in five lily species.
