# Supplementary figures and images for: Comprehensive Analysis of Secondary Metabolites in the Extracts from Different Lily Bulbs and Their Antioxidant Ability
Source: Antioxidants (Basel). 2021 Oct 17;10(10):1634. doi: 10.3390/antiox10101634 (PMC8533310; doi:10.3390/antiox10101634)

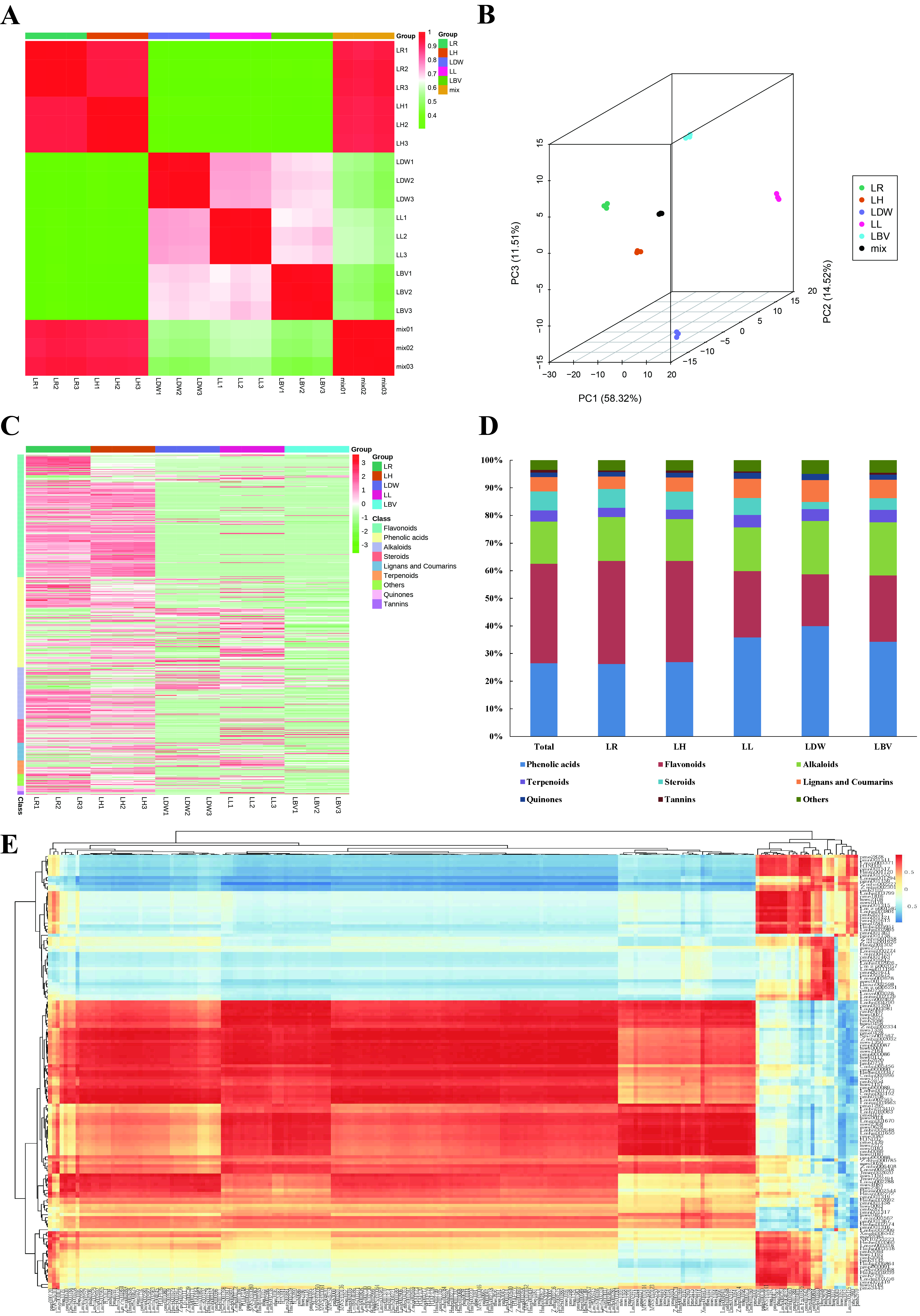

Supplement: Supplementary file 1 [file antioxidants-10-01634-s001.zip › Figure S1.jpg]

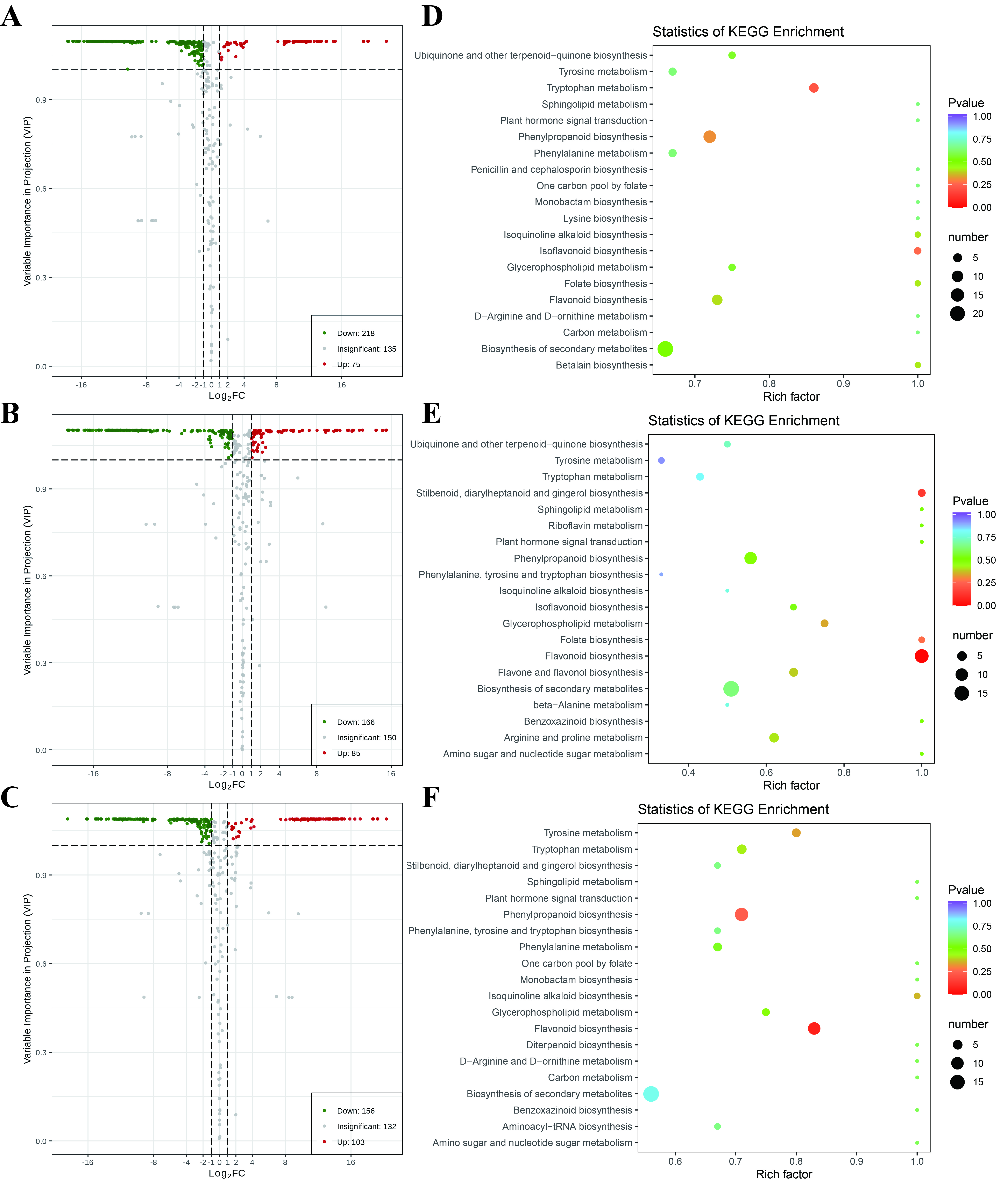

Supplement: Supplementary file 1 [file antioxidants-10-01634-s001.zip › Figure S2.jpg]
